# Experimental and Computational Study of Ductile Fracture in Small Punch Tests

**DOI:** 10.3390/ma10101185

**Published:** 2017-10-17

**Authors:** Betül Gülçimen Çakan, Celal Soyarslan, Swantje Bargmann, Peter Hähner

**Affiliations:** 1 Department of Mechanical Engineering, Uludağ University, Görükle, 16059 Bursa, Turkey; 2 Chair of Solid Mechanics, School of Mechanical Engineering and Safety Engineering, University of Wuppertal, 42119 Wuppertal, Germany; soyarslan@uni-wuppertal.de (C.S.); bargmann@uni-wuppertal.de (S.B.); 3 Nuclear Safety and Security Directorate, Joint Research Centre, European Commission, NL-1755 LE Petten, The Netherlands; peter.haehner@ec.europa.eu

**Keywords:** small punch test, P91 steel, ductile fracture, gurson’s plasticity model, nonlocal plasticity

## Abstract

A unified experimental-computational study on ductile fracture initiation and propagation during small punch testing is presented. Tests are carried out at room temperature with unnotched disks of different thicknesses where large-scale yielding prevails. In thinner specimens, the fracture occurs with severe necking under membrane tension, whereas for thicker ones a through thickness shearing mode prevails changing the crack orientation relative to the loading direction. Computational studies involve finite element simulations using a shear modified Gurson-Tvergaard-Needleman porous plasticity model with an integral-type nonlocal formulation. The predicted punch load-displacement curves and deformed profiles are in good agreement with the experimental results.

## 1. Introduction

Small punch (SP) testing is used at high homologous temperatures for investigation of creep properties such as rupture time and minimum creep rate or at low homologous temperatures for investigation of other mechanical properties such as yield stress, ultimate stress or fracture toughness. The test requires smaller specimen sizes as compared to standard mechanical tests. Hence, it allows investigations of regions with gradients properly, such as heat affected zones [1,2]. Due to its small size the test is almost non-destructive. Thus, it eliminates the need for repairing the component after sample removal, which is another advantage of the SP test compared to the standard tests. During an SP test the sample deforms by different deformation mechanisms initially by elastic and plastic bending and followed by membrane stretching. Hence, a rather complex multiaxial stress state, evolving with puncher displacement, occurs inside the sample. The sample thickness closely affects the occurrence of these deformation modes. If the thickness is increased then shearing mode becomes more dominant as compared to membrane stretching [3]. In a recent work [4], the authors applied Gurson-Tvergaard-Needleman/Ritchie-Knott-Rice (GTN/RKR) approach to plain and notched SP disks for a wide temperature range. The current study aims at a detailed investigation of deformation mechanisms as well as crack initiation and propagation at room temperature SP tests of P91 steel with different disk thicknesses. To this end, the problem is studied by experimental and numerical analyses in order to:

- Further exploit multiaxial SP testing for numerical model validation,
- Consolidate the parameter identification through application of the model to a wide range of disk thicknesses,
- Elucidate the effects of shear and initial porosity on damage progression and failure,
- Derive robust predictions for the yield stress (YS) and ultimate tensile stress (UTS) for future applications.

On the experimental side, SP tests are carried out using disks of P91 material which is a heat resistant steel widely used in power plants. Due to this reason it is frequently preferred as the test material in the SP method [1,2,5,6]. The SP disks are produced with varying thicknesses from 0.2 mm to 1 mm were tested up to complete fracture. At all thicknesses, due to lack of notch, large scale yielding conditions prevail (at least before intense localization with voidage) and the observed consequent fracture mode is ductile. For smaller thicknesses the fracture is led by diffuse necking followed by an intense localization throughout the section. For larger thicknesses, on the other hand, an initial diffuse necking pattern is not observed since shear deformation prevails rather than membrane stretching. Unlike a simple tension test where the cracks start inside the specimen, the crack initiates from the bottom surface at a distance where thinning takes place. As will be shown, with increasing thickness the fracture occurs at larger punch displacements regarding both the computational and experimental curves. Scanning electron microscope (SEM) studies of the fracture surfaces show signs of a mixed mode I (normal) mode II (shear) fracture with relatively flat dimple walls elongated along the fracture surface. Fracture is primarily due to voidage nucleated at M_23_C_6_ carbides and/or MX precipitates coalesced under severe shearing.

On the computational side, finite element simulations are carried out for the given thickness range. To this end a shear modified GTN model with strain hardening and strain rate hardening is implemented to model the ductile fracture based on void nucleation, growth and coalescence. The origin of the model goes back to Gurson [7] and later modifications were introduced by Tvergaard and Needleman to better account for cavity growth [8,9,10]. More recently, a shear modification has been proposed in [11] to accommodate softening by inter-void linking under low stress triaxiality conditions which is not accounted for in the original formulation. Pathological mesh sensitivity pertaining to softening is remedied by an integral-type nonlocal formulation applied to each additive void volume fraction rate component similar to [12]. This formulation requires a characteristic length hence allowing incorporating the size effect in the model. This model is implemented as a Vumat user defined material subroutine for Abaqus/Explicit. Prior to applications to the SP test, the implementation is validated using single element tests under dilatation and simple shear conditions for which analytical solutions exist. The effectiveness of the implemented delocalization scheme is also demonstrated by plane strain tensile tests with different mesh sizes. These studies are followed by SP simulations with 2D axisymmetric models. Model parameters for P91 steel including the associated length scale are identified using quantitative measurements, i.e., metallographic images as well as inverse methods relying on experimentally determined force-displacement curves. A good agreement of not only the force-displacement curves but also the deformed profiles of the failed samples in between the computationally and experimentally determined results is obtained. A parameter sensitivity analysis is also conducted where the influence of shear damage parameter $k_{w}$ and the initial porosity $f_{0}$ are investigated. Since the gradient of solution dependent fields are relatively low during the tests in these investigations, the effect of nonlocal regularisation is not observed until the onset of localization. It is believed that the nonlocal modeling framework developed will be helpful to further analyze the effects of strain rate, specimen size, puncher head geometries and especially notched specimens where higher gradients of field variables could prevail.

## 2. Theory

### 2.1. A Word on Notation

Assuming a, b and c as three second order tensors, together with the Einstein’s summation convention on repeated indices, c=a·b represents the single contraction product with $c_{ik}=a_{ij}b_{jk}$. d=a:b represents the double contraction product with $d=a_{ij}b_{ij}$, where *d* is a scalar. E=a⊗b, F=a⊕b, and G=a⊖b represent the tensor products with $E_{ijkl}=a_{ij}b_{kl}$, $F_{ijkl}=a_{ik}b_{jl}$, and $G_{ijkl}=a_{il}b_{jk}$, where E, F, and G represent fourth order tensors. $a^{⊤}$ and $a^{-1}$ denote the transpose and the inverse of a, respectively. $\partial_{a}b$ denote ∂b/∂a. $\mathrm{dev}\left(a\right)$ and $\mathrm{tr}\left(a\right)$ stand for the deviatoric part of and trace of a, respectively, where $\mathrm{dev}\left(a\right)=a-\left[1/3\right]$tr$\left(a\right)1$, with tr$\left(a\right)=a_{ii}$ and 1 denoting the identity tensor. $\mathrm{sym}\left(a\right)$ and $\mathrm{skw}\left(a\right)$ denote symmetric and skew-symmetric portions of a. $\dot{a}$ gives the material time derivative of a. $\langle x\rangle =\left[1/2\right]\left[x+\left|x\right|\right]$. Finally, $\hat{a}$ gives a represented at the rotationally neutralized configuration.

### 2.2. Fundamental Kinematical Assumptions and Hypoelastic Plasticity

Let $F:=\partial_{X}x$ define the deformation gradient of the nonlinear map $\varphi :\Omega_{0}\times R\to R^{3}$ where X and x:=φ(X,t) denote the particle positions at the reference (undeformed) configuration $\Omega_{0}$ and current (deformed) configuration Ω respectively. Then, $l:=\dot{F}\cdot F^{-1}=\partial_{x}v$ denotes the spatial velocity gradient, with $v=\dot{x}$. An additive split of $d:=\mathrm{sym}\left(l\right)$ into elastic and plastic parts (Various forms of rate additive splits can be recovered through a multiplicative decomposition of F into elastic $F^{e}$ and plastic $F^{p}$ parts with $F:=F^{e}\cdot F^{p}$ making use of $\dot{F}={\dot{F}}^{e}\cdot F^{p}+F^{e}\cdot {\dot{F}}^{p}$ and ${\left[F\right]}^{-1}={\left[F^{p}\right]}^{-1}\cdot {\left[F^{e}\right]}^{-1}$. Accordingly one has $l=l^{e}+l^{p}$ where $l^{p}\to F^{e}\cdot L^{p}\cdot {\left[F^{e}\right]}^{-1}$ with $l^{e}:={\dot{F}}^{e}\cdot {\left[F^{e}\right]}^{-1}$ and $L^{p}:={\dot{F}}^{p}\cdot {\left[F^{p}\right]}^{-1}$. For small elastic strains and rigid body rotations with $F^{e}≃1$ one governs $l^{p}\to L^{p}$. Using $F^{e}:=R^{e}\cdot U^{e}$ and assuming small elastic strains but finite rotations, i.e., $U^{e}≃1$, supplies $l^{p}\to R^{e}\cdot L^{p}\cdot {\left[R^{e}\right]}^{-1}$. Taking the symmetric part of both sides one reaches $d=d^{e}+d^{p}$. A small elastic strain assumption makes a hypoelastic constitutive equation in terms of $d^{e}$ plausible.) is hypothesised to reach $d=d^{e}+d^{p}$. We then introduce the rotationally neutralized rate of deformation tensor $\dot{\hat{\epsilon }}$ defined as

(1) $$\dot{\hat{\epsilon }}={\dot{\hat{\epsilon }}}^{e}+{\dot{\hat{\epsilon }}}^{p},$$

with ${\dot{\hat{\epsilon }}}^{e}:=R^{⊤}\cdot d^{e}\cdot R$ and ${\dot{\hat{\epsilon }}}^{p}:=R^{⊤}\cdot d^{p}\cdot R$. Here, R is the rotation tensor found through the polar decomposition of the deformation gradient with F=R·U. Similarly, a pull back operation on the Cauchy stress tensor σ with the rotation tensor gives its rotationally neutralized counterpart viz $\hat{\sigma }:=R^{⊤}\cdot \sigma \cdot R$ whose material time derivative $\dot{\hat{\sigma }}$ is postulated to obey the following hypoelastic relation

(2) $$\dot{\hat{\sigma }}=C^{e}:{\dot{\hat{\epsilon }}}^{e},$$

with $C^{e}:=\left[K-2\mu /3\right]\left[1⊗1\right]+\mu \left[1⊕1+1⊖1\right]$ where *K* and μ denote the bulk and the shear modulus, respectively.

### 2.3. Shear Modified GTN Porous Plasticity—Local Formulation

The model used here is Gurson’s dilatant plasticity model [7], which is extended by parameters $q_{1}$, $q_{2}$ and $q_{3}$ in [8,9] to account for a better agreement with the computational analyses of various void distributions, and by the bilinear function $f^{*}\left(f\right)$ in [10] to account for rapid void coalescence prior to failure. The hydrostatic stress dependent flow potential $\Phi^{p}$ is then formulated as

(3) $$\Phi^{p}={\left[\frac{\sigma_{eq}}{\sigma_{y}}\right]}^{2}+2q_{1}f^{*}\cosh\left(\frac{3}{2}\frac{q_{2}\sigma_{m}}{\sigma_{y}}\right)-\left[1+q_{3}f^{*2}\right]=0\;.$$

with

(4) $$f^{*}\left(f\right)=\left\{\begin{array}{cc} f & f\leq f_{c}\;,\\ f_{c}+\left[f_{u}^{*}-f_{c}\right]\left[f-f_{c}\right]/\left[f_{f}-f_{c}\right] & f>f_{c}\;. \end{array}\right.$$

where $\sigma_{eq}$ is the (macroscopic) equivalent von Mises stress, $\sigma_{y}$ is the (matrix) yield stress, $\sigma_{m}$ is mean stress, *f* is the void volume fraction and $q_{1}$, $q_{2}$ and $q_{3}$ are material parameters. $f_{c}$ denotes the critical void volume fraction at incipient coalescence, $f_{f}$ the fraction at failure and $f_{u}^{*}=1/q_{1}$.

The viscoplastic hardening of the material matrix is described by $\sigma_{y}$ which accounts for strain and strain rate dependence. Hence, letting $e^{p}$ denote the equivalent plastic strain and ${\dot{e}}^{p}$ its rate, we assume the following multiplicative form

(5) $$\sigma_{y}\left(e^{p},{\dot{e}}^{p}\right)=h_{y}\left(e^{p}\right)r_{y}\left({\dot{e}}^{p}\right)\;,$$

where $h_{y}$ and $r_{y}$ respectively denote the functions of strain hardening and strain rate hardening

(6) $$\left.\begin{array}{ccc} h_{y}\left(e^{p}\right) & = & \left\{\begin{array}{cc} \sigma_{y0}+h_{0}e^{p} & e^{p}\leq e_{0}^{p}\\ h_{1}e^{p}+\sigma_{y\infty }-\left[\sigma_{y\infty }-\sigma_{y1}\right]\exp\left(-m{\left[e^{p}\right]}^{n}\right) & e^{p}>e_{0}^{p} \end{array}\right.,\\ r_{y}\left({\dot{e}}^{p}\right) & = & 1+C\log\left({\dot{e}}^{p}/{\dot{e}}_{0}^{p}\right)\;, \end{array}\right\}$$

where $\sigma_{y0}$, $\sigma_{y1}$, $\sigma_{y\infty }$, $h_{0}$, $h_{1}$, *m*, *n* and $e_{0}^{p}$ are plastic strain hardening parameters. *C* and ${\dot{e}}_{0}^{p}$, on the other hand, are parameters controlling the rate dependence.

Associated plastic flow rule gives the plastic rate of deformation tensor at the rotationally neutralized configuration as

(7) $${\dot{\hat{\epsilon }}}^{p}=\dot{\gamma }\partial_{\hat{\sigma }}\Phi^{p}\;,$$

where $\dot{\gamma }$ denotes the plastic multiplier. The equivalent plastic strain rate, using the plastic work equivalence with $\left[1-f\right]\sigma_{y}{\dot{e}}^{p}=\hat{\sigma }:{\dot{\hat{\epsilon }}}^{p}$, reads

(8) $${\dot{e}}^{p}=\frac{\hat{\sigma }:{\dot{\hat{\epsilon }}}^{p}}{\left[1-f\right]\sigma_{y}}\;.$$

The void volume fraction evolution involves nucleation and growth. The rate of the total void volume fraction is formulated additively in terms of void nucleation rate ${\dot{f}}^{n}$ and void growth rate ${\dot{f}}^{g}$

(9) $$\dot{f}={\dot{f}}^{n}+{\dot{f}}^{g}.$$

For ${\dot{f}}^{n}$ we assume a strain dependent void nucleation [13]

(10) $${\dot{f}}^{n}=A_{N}{\dot{e}}^{p}\;\;\mathrm{where}\;A_{N}=A_{N}\left(e^{p}\right)=\frac{f_{N}}{S_{N}\sqrt{2\pi }}\exp\left(-\frac{{\left[e^{p}-e_{N}^{p}\right]}^{2}}{2{\left[S_{N}\right]}^{2}}\right)\;,$$

where $e_{N}^{p}$ and $S_{N}$ respectively denote the mean equivalent plastic strain at the incipient nucleation and its standard deviation. $f_{N}$ denotes the total source for void volume fraction of nucleation. ${\dot{f}}^{g}$ is further split into two parts [11]

(11) $${\dot{f}}^{g}={\dot{f}}_{\mathrm{normal}}^{g}+{\dot{f}}_{\mathrm{shear}}^{g}\;,$$

where ${\dot{f}}_{\mathrm{normal}}^{g}$ accounts for the void growth under hydrostatic stresses whose formulation simply uses the mass conservation viz.

(12) $${\dot{f}}_{\mathrm{normal}}^{g}=\left[1-f\right]\mathrm{tr}\left({\dot{\hat{\epsilon }}}^{p}\right)\;,$$

whereas ${\dot{f}}_{\mathrm{shear}}^{g}$ accounts for softening effects associated with void distortion and void interaction with material rotation under shear stress states where

(13) $${\dot{f}}_{\mathrm{shear}}^{g}=k_{w}f\;w\left(\mathrm{dev}\hat{\sigma }\right)\;\frac{{\dot{\hat{\epsilon }}}^{p}:\mathrm{dev}\;\hat{\sigma }}{\sigma_{eq}}\;.$$

Here, $k_{w}$ is a material parameter which scales shear damage rate with a suggested interval $0\leq k_{w}\leq 3$, [11]. $w\left(\mathrm{dev}\;\hat{\sigma }\right)=1-{\left[27J_{3}/2\sigma_{eq}^{3}\right]}^{2}$ with 0≤w≤1 distinguishes the states of axisymmetric stress from those of generalized shear on the Π−plane. Here, w=0 through $J_{3}=\pm \left[2/27\right]\sigma_{eq}^{3}$ for all axisymmetric stress states, whereas w=1 through $J_{3}=0$ for the states of generalized shear. This modification due to [11] is motivated by the reported experimental evidence for low triaxiality fracture development in, e.g., [14,15], for which the original GTN model falls short in predictive capability.

**Figure 1:**
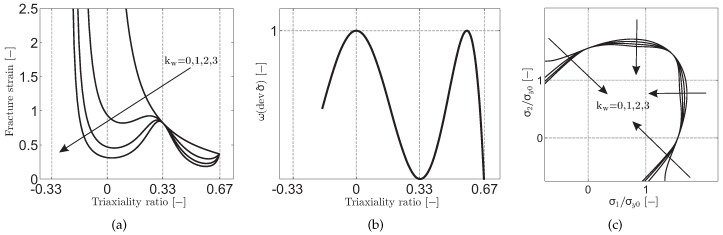
(**a**) The influence of the shear damage parameter $k_{\omega }$ on the dependence of fracture strain on triaxiality ratio; (**b**) The plot of the function $w(\mathrm{dev}\;\hat{\sigma })$; (**c**) Isochronous fracture surfaces for varying $k_{\omega }$ in plane stress space. These plots are created by numerically solving the initial boundary value problem for linear and uniform strain paths under plane stress state considering linear isotropic hardening plasticity with hypothetical parameters.

The fracture responses for the shear modified Gurson model are given in Figure 1 for linear and uniform strain paths under plane stress state considering linear isotropic hardening plasticity with hypothetical parameters. As the figure clearly depicts the monotonic dependence of the fracture strain on the stress triaxiality is suppressed for $k_{w}\neq 0$. Further, there occurs considerable shrinkage in the admissible range of deformation for generalized plane strain paths, i.e., pure shear and plane strain loading paths with vanishing stress triaxiality ratio $\sigma_{m}/\sigma_{y}\to 0$. On the other hand, strain paths associated with the axisymmetric stress states, i.e., uniaxial and biaxial loading paths respectively with $\sigma_{m}/\sigma_{y}=1/3$ and $\sigma_{m}/\sigma_{y}=2/3$, are not affected by the shear correction since for these paths the shear fracture controlling parameter $w(\mathrm{dev}\;\hat{\sigma })$ becomes zero with vanishing third invariant of the deviatoric stress tensor: $J_{3}=0$. Applications of the model to formability exhaustion of DC04 steels during sheet-bulk forming and to the problem of severe plastic localization bands initiated at free surfaces during free bending are given in [16,17], respectively.

#### Integral-Type Nonlocal Extension

The motivation behind integral-type nonlocal formulations is twofold. On the mechanical side, with micro-void and micro-crack interactions being their micromechanical motivation, integral-type nonlocal formulations constitute distributed damage models capable of reproducing size effects. In view of finite element analysis with damage models, they remedy the pathological mesh dependence of the local solution where the size of the process zone and associated energy dissipation per unit volume is dictated by the discretization. With this motivation, in the current study, an integral-type nonlocal formulation is adopted which relies on the following delocalization operation

(14) $$v_{\mathrm{nonlocal}}\left(x\right)=\int_{V}\tilde{\omega }\left(x,y\right)v\left(y\right)dV\left(y\right)\;.$$

Here y represents the location vector and *V* the volume at the current coordinates. If $\omega \left(x,y\right)$ denotes the bell-shaped nonlocal weight function

(15) $$\omega \left(x,y\right)=\left\{\begin{array}{cc} {\left[{1-|x-y|}^{2}/R^{2}\right]}^{2} & \;\mathrm{if}\;|x-y|\leq R,\\ 0 & \;\mathrm{otherwise}, \end{array}\right.$$

the normalized weight function $\tilde{\omega }\left(x,y\right)$, which remedies any inconsistency pertaining to the unrestricted averaging domains extending over the problem boundary, reads

(16) $$\tilde{\omega }\left(x,y\right)=\frac{\omega \left(x,y\right)}{\int_{V}\omega \left(x,y\right)dV\left(y\right)}\;.$$

As long as the boundaries are not violated $\int_{V}\omega \left(x,y\right)dV\left(y\right)$ is a constant. *R* in Equation (15) denotes the interaction radius which constitutes the characteristic length. For R→0 a local formulation is recovered. In practical applications, *R* is related to the material microstructure, e.g., four times void size or half void spacing for ductile fracture mechanism [18]. Delocalization could be applied either directly to the kinematic fields; see, e.g., [19], or to the damage variables which control softening, see, e.g., [12,18,20,21]. In the current study, delocalization of each additive rate component of the void volume fraction is applied. Hence, *v* in (14) is substituted by the damage rate component as follows

(17) $$\left.\begin{array}{ccc} {\dot{f}}_{\mathrm{nonlocal}}^{n}\left(x\right) & = & \int_{V}\tilde{\omega }\left(x,y\right){\dot{f}}^{n}\left(y\right)dV\left(y\right)\;,\\ {\dot{f}}_{\mathrm{normal},\;\mathrm{nonlocal}}^{g}\left(x\right) & = & \int_{V}\tilde{\omega }\left(x,y\right){\dot{f}}_{\mathrm{normal}}^{g}\left(y\right)dV\left(y\right)\;,\\ {\dot{f}}_{\mathrm{shear},\;\mathrm{nonlocal}}^{g}\left(x\right) & = & \int_{V}\tilde{\omega }\left(x,y\right){\dot{f}}_{\mathrm{shear}}^{g}\left(y\right)dV\left(y\right)\;, \end{array}\right\}$$

which finally adds up to the total nonlocal porosity rate ${\dot{f}}_{\mathrm{nonlocal}}$, viz.

(18) $${\dot{f}}_{\mathrm{nonlocal}}={\dot{f}}_{\mathrm{nonlocal}}^{n}+{\dot{f}}_{\mathrm{normal},\;\mathrm{nonlocal}}^{g}+{\dot{f}}_{\mathrm{shear},\;\mathrm{nonlocal}}^{g}.$$

One notes that for spatially uniform porosity rate distributions delocalization is not effective. Hence, local and nonlocal theories differ only if field gradients exist. The material model is implemented as a Vumat material subroutine for Abaqus/Explicit. The algorithmic details are given in the supplementary materials. The natural setting of Vumat alone is not appropriate for nonlocal formulations and it allows only local effects to be modeled. To this end, in our treatment we have additionally implemented a Vusdfld routine. 

## 3. Experiments

### 3.1. Material Employed

**Figure 2:**
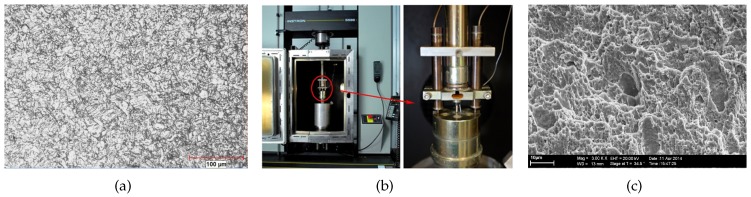
(**a**) Optical micrograph of the tempered martensitic structure of P91 steel; (**b**) Experimental setup for SP testing; (**c**) Fracture surface of the unnotched 0.5mm thick SP disk showing the dimpled structure.

The material used in this study is modified P91 which has a tempered martensitic microstructure. An optical image revealing the microstructure is given in Figure 2a. The prior austenite grain size was approximately 14–25 μm. The chemical composition of the material is (wt %) 0.10 C, 0.27 Si, 0.53 Mn, 0.007 P, 0.01 S, 8.76 Cr, 0.91 Mo, 0.2 V, 0.35 Ni, 0.04 Nb, 0.038 N. Conventional disk shaped SP specimens with a diameter of 8 mm and initial thickness in the range of 0.2 mm to 1 mm were used. Attention was paid not to violate the tolerances stated for disk thicknesses (±1% of nominal thickness) during the polishing process.

### 3.2. Experimental Setup

SP tests were performed using the experimental setup in Figure 2b. The major components of the set up are a specimen holder to ensure the tight clamping of the SP disk, a puncher with a hemispherical head of 2.5 mm diameter for the central loading of the disk and two Linear Variable Displacement Transducers to measure the displacement of the puncher. The SP specimens were circumferentially clamped to prevent slippage of the SP specimen during the test. The aperture of the receiving die was 4 mm in diameter with an edge chamfer of 0.2 mm. The crosshead displacement rate was 0.005 mm/s. All the tests were carried out at room temperature (25 °C) in accordance with the Code of Practice for Small Punch Testing [22]. The typical output from an SP test is a force-displacement curve.

### 3.3. Crack Propagation and Fracture Surfaces

During SP testing at room temperature, the fracture is ductile and proceeded by uniform necking. The crack initiates from the bottom surface at a distance where necking takes place. Then, it propagates in direction of the maximum equivalent plastic strain through the thickness and follows a circumferential path along this necking region [23,24]. This is valid regardless of disk thickness.

For most metals, voids nucleate from inclusions and secondary phase particles by particle cracking or interface decohesion with increasing plastic strain. If particles are not large like MnS inclusions, the voids nucleate by debonding of the particle-matrix interface and grow with the plastic deformation in the matrix. The resulting fracture surface exhibits a dimpled structure with many microvoids. In Figure 2c the fracture surface of the SP disk with 0.5 mm thickness is presented. This image reveals a dimpled fracture surface consisting of high density of small microvoids and lower density of relatively large and deep ones. The approximate distance between the large dimples is found to be 30–35 μm whereas the distance was 2–5 μm for the small dimples. The initiation of small microvoids is attributed to MX precipitates distributed in the matrix which are higher in density. The larger microvoids presumably are initiated by larger M_23_C_6_ carbides which are mainly precipitated on the grain and lath boundaries. The alignment of the dimple walls shows that fracture does not take place with void growth under Mode I, in which one would expect dimple wall elongation orthogonal to the surface, but a mixed Mode I and Mode II, where shearing is incorporated. The shearing direction acutely angled with the vertical is distinguishable from the fractograph of Figure 2c.

## 4. Material Parameters for P91 Steel

The elastic bulk modulus of P91 at room temperature is K= 175,000 MPa and the shear modulus is G= 80,769 MPa. The isotropic hardening plasticity parameters are given in Table 1. Since no rate sensitivity is observed for P91 in the conditions of interest, the corresponding parameters are arranged to suppress the rate effect.

Letting $d_{x},$$d_{y}$ and $d_{z}$ denote average inclusion diameters in the respective directions and S(%) and Mn (%) represent the weight percentages of sulphide and manganese in the matrix, respectively, the initial porosity can be estimated using quantitative metallography from Franklin’s formula [25].

(19) $$f_{0}=\frac{0.054\sqrt{d_{x}d_{y}}}{d_{z}}\left[S\left(\%\right)-\frac{0.001}{\mathrm{Mn}\left(\%\right)}\right].$$

Assuming approximately spherical inclusions ($d_{x}=d_{y}=d_{z}$), the initial porosity is calculated as $f_{0}=0.00044$, based on the previously given chemical properties of P91. A higher value of $f_{0}$ was suggested ($f_{0}=0.002$) in [26] thus in further sections the effect of $f_{0}$ on simulation results was investigated by using $f_{0}=0.00044$ and $f_{0}=0.002$. Franklin’s formula serves as an estimate for the initial void volume fraction $f_{0}$. The authors’ simulations on unnotched disks show that initial porosity estimates within (0.0044, 0.002) resulted in only marginal differences in the computed failure times. Regarding the void nucleation, depending on the bimodal distribution of particles, it is possible to postulate two separate strain dependent probability distribution for each nucleation source. This requires mode detailed analysis which are not available to the authors. Hence, for the void nucleation kinetics we content with using previously suggested material parameters in the literature. In this context, we believe that separation nucleation mechanisms requires further studies. The parameters $q_{1}=1.5$, $q_{2}=1$, $q_{3}=q_{1}^{2}$ of the extended Gurson model are chosen following [8,9]. Motivated by the fact that the volume fraction of the segregated inclusions $f_{N}$ is within a narrow band of 0.01 to 0.03 the parameters controlling void nucleation are chosen as $f_{N}=0.02$, $\epsilon_{N}=0.3$ and $S_{N}=0.1$ [27,28]. The proposed range of $k_{w}$ for structural alloys is reported as $0<k_{w}<3$, see, e.g., [11]. In the current study, $k_{w}=0$ gives the best results for the predicted fracture strains. Nevertheless, a sensitivity analysis is performed investigating the effect of $k_{w}$ for various disk thicknesses.

Pertaining to the void coalescence and final fracture, the European Structural Integrity Society round robin [29] recommends the slope of the tail of the bilinear coalescence function (6) as 4. For the final void volume fraction at failure different references give different results, e.g., [30] takes $f_{f}=0.25$ in accordance with [31] whereas $f_{f}=0.2$ is used in [27,28,32,33], where the last two studies refer to steel A533 B. Based on the room temperature parameter fitting studies for P91 steels the coalescence and failure porosity are taken as $f_{c}=0.1$ and $f_{f}=0.25$.

Applying a nonlocal regularisation to local formulation of porous plasticity, introduction of a characteristic length scale is necessary. This length scale has been related to a physical quantity such as (four times) void size or (half) void spacing for ductile fracture mechanism [18]. If two populations of second phase particles are present, which is the case for P91, one should select the population which is dominant in crack initiation and propagation. While Xia and Shih observed that large inclusions constitute the main contribution to void formation while small inclusions only assist the hole link-up [34]. Thus, they selected the length scale as the mean spacing between voids nucleated by large inclusions. As seen from the fracture surface in Figure 2c, the small voids nucleated by MX precipitates are dominating in quantity and the mean distance between them ranges from 1.5 μm to 2.5 μm. On the other hand, the distance between large voids nucleated by M_23_C_6_ precipitates are between 20 μm and 30 μm. Regarding the two studies mentioned, we use R=5 μm as an average value considering the two population of precipitates. This value is also validated by the simulations.

## 5. Small Punch Test Simulations

### 5.1. Finite Element Model

2D axisymmetric simulations with CAX4R reduced integration elements for room temperature are conducted in Abaqus/Explicit with double precision. A solution of quasi-static problems with a dynamic-explicit solution procedure generally involves a very large number of time steps. In order to reduce the computational cost, mass scaling is applied with a target time step of $10^{-3}$ s over the whole analysis which lasts for 150 s. This supplies acceleration of the simulations without changing the actual time scale of the process. The dies and the punch are modeled as rigid bodies and the disk as a deformable body. The interaction in-between the rigid and deformable bodies is assumed to be constant with a dynamic friction coefficient chosen as μ=0.25, which is taken to be temperature independent. The friction coefficient has a direct influence on the necking as well as damage initiation location. In order to show this more clearly we have conducted a new set of simulations with and without friction effects. We have observed that the location of the localization gets closer to the center as the friction coefficient decreases. The thinning at the localization is more apparent with higher coefficients whereas it is less at the center. It is observed from the force-displacement curves that the crack initiation and failure takes place at lower displacements if the friction effect is higher due to the more pronounced thinning at the necking location.

**Figure 3:**
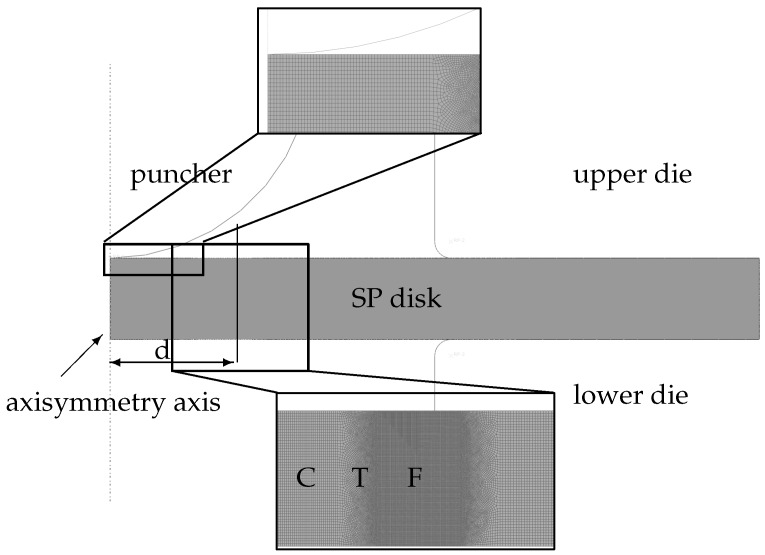
Axisymmetric finite element model and the mesh used for 0.5 mm thick disk: F-Fine, T-Transition, C-Coarse Mesh Regions. The width of the T + F is approximately 0.6 mm and the distance of this region from the center of the disk, d, is 0.7 mm. (minimum element size: 1.5 × 2.5 μm, number of elements: 59,143). The same meshing pattern is applied to the disks with other thicknesses.

The mesh used for 0.5 mm thickness is given in Figure 3. If using an integral-type nonlocal formulation, the element size should be below the characteristic length. For R=5 μm which is selected depending on the microstructure of P91, this requirement puts severe restrictions on the selected mesh size. In order to reduce the computational time non-uniform meshing was used for the model: refined mesh at the crack location, coarse mesh at the clamped part. Initially, a uniform coarse mesh is applied to identify the approximate crack location. Then, refined meshing is applied to this region of interest.

Crack propagation is modeled using an element erosion technique: elements with Gauss points whose damage reaches the corresponding failure thresholds are removed from the computational stack. Although the applied nonlocal formulation removes the mesh dependence of the field distributions to an extent, for the crack propagation this could be limited. Hence, since element erosion is used, the direction of the crack and the crack propagation size are controlled by the mesh. Once biased and/or coarse mesh is used, predicted crack paths will be inevitably poor. In this study, sufficiently small sized elements with irregular distributions are used. The irregularity of the mesh created by the advancing front quad method accounts for the microstructural heterogeneity. With the same motivation the number of elements within the effective radius of each element vary spatially.

### 5.2. Results and Discussion

#### 5.2.1. The Influence of Geometrical Parameters: Thickness

During SP test as the puncher deforms the SP specimen, multi-axial stress and strain fields occur. Various deformation stages take place: elastic bending, plastic bending, membrane stretching and, eventually, crack initiation and failure of the specimen.

**Figure 4:**
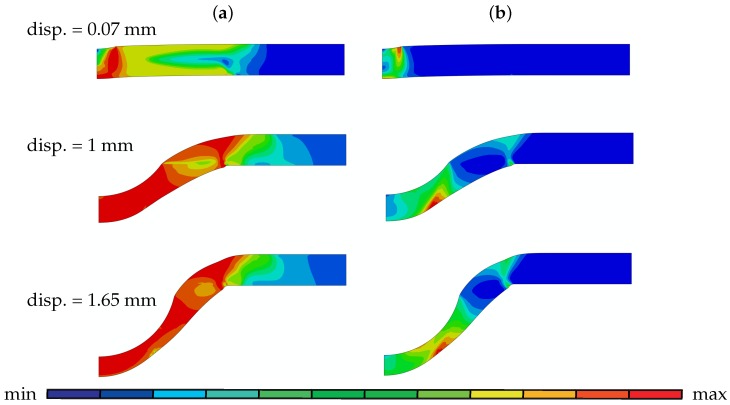
(**a**) von Mises stress and (**b**) plastic strain plots of SP disk with 0.5 mm thickness at various displacements: 0.77 mm (**a**) [max $7.69\times 10^{2}$; min 0], (**b**) [max $1.24\times 10^{-1}$; min 0], 1 mm (**a**) [max $8.56\;\times \;10^{2}$; min 0], (**b**) [max $6.83\times 10^{-1}$; min 0] and 1.65 mm (**a**) [max $8.71\times 10^{2}$; min 0], (**b**) [max 1.12; min 0].

The von Mises stress plots (Figure 4) of the SP disk with 0.5 mm thickness reveals that with the onset of contact between the puncher and the disk, high stresses occur underneath the contact region and this zone expands with the contact region till the crack initiation. Due to high stresses developed along with the contact, plastic deformation takes place underneath the contact region at the beginning of the test. As the puncher continues to deform the disk, maximum plastic deformation moves to the bottom surface and localizes in this region where crack initiates.

**Figure 5:**
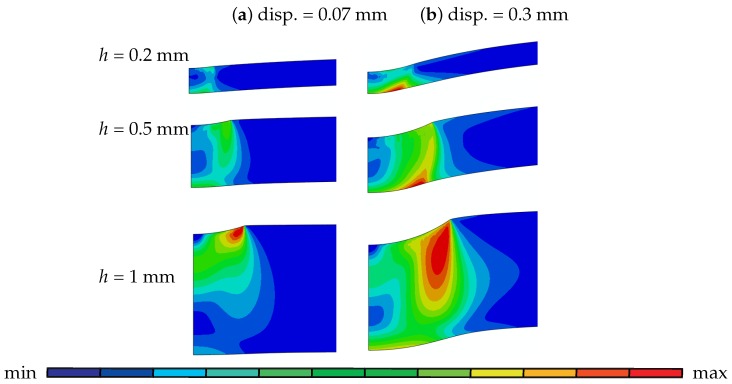
Plastic indentation underneath the puncher at the initial stage of the test according to disk thicknesses: at disp = 0.07 mm for *h* = 0.2, 0.5 and 1 mm [max $1.98\times 10^{-1}$; min 0], at disp = 0.3 mm for *h* = 0.2 mm [max $2.11\times 10^{-1}$; min 0], for *h* = 0.5 mm [max $2.64\times 10^{-1}$; min 0] and for *h* = 1 mm [max $3.03\times 10^{-1}$; min 0].

The deformation modes are intimately dependent on the specimen thickness. For thinner specimens, considerable thickness change and stretching occurs during the test until fracture. On the other hand, indentation caused by the localized plastic yielding underneath the puncher gets more pronounced with increasing thickness. This is revealed by the plastic strain plots of 0.2 mm, 0.5 mm and 1 mm disks at the initial stage (at displacement ∼0.07 mm and 0.3 mm) of the test given in Figure 5. The plastically deformed region under the contact area is more pronounced for the 1 mm thick disk compared to the thinner disks of 0.2 mm and 0.5 mm. As the puncher further penetrates through the disk, the plastically strained region for the 1 mm disk expands in the upper surface and moves away from the center, whereas for 0.2 mm and 0.5 mm it localizes at the bottom surface.

**Figure 6:**
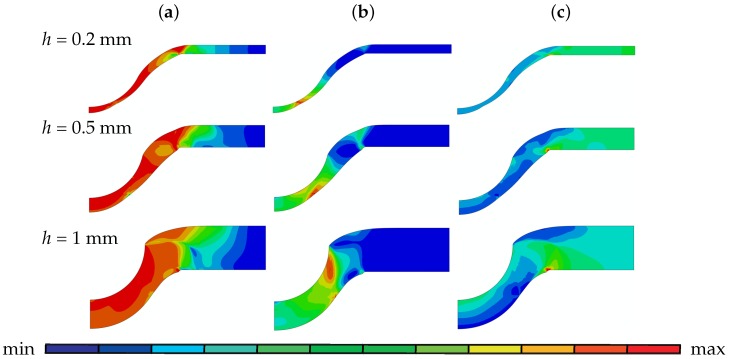
Field distributions before crack initiation: (**a**) von Mises Stress; (**b**) plastic strain and (**c**) pressure for disks with 0.2 mm, 0.5 mm and 1 mm thickness: for *h* = 0.2 mm (**a**) [max $8.30\times 10^{2}$; min $1.18\times 10^{2}$], (**b**) [max 1.10; min 0], (**c**) [max $1.33\times 10^{3}$; min $-8.87\times 10^{2}$], for *h* = 0.5 mm (**a**) [max $8.70\times 10^{2}$; min $6.68\times 10^{1}$], (**b**) [max 1.14; min 0], (**c**) [max $1.49\times 10^{3}$; min $-7.22\times 10^{2}$], and for *h* = 1 mm (**a**) [max $9.13\times 10^{2}$; min $2.18\times 10^{1}$], (**b**) [max 1.20; min 0], (**c**) [max $1.39\times 10^{3}$; min $-6.21\times 10^{2}$].

In order to make further evaluations to reveal the influence of the disk thickness, we present the von Mises stress, plastic strain, pressure plots of 0.2 mm, 0.5 mm and 1 mm thick disks prior to crack initiation in Figure 6. For all thicknesses, maximum plastic strain occurs at the bottom surface of the disk where tensile stresses prevail and promote voidage. For the 1 mm disk there is also a highly strained region at the upper side close to the clamped region which occurs under the combined influence of tension and shear. Again for all thicknesses, the crack initiation starts from the bottom surface where plastic strain is maximum. Practically, the whole unclamped part of the disk exhibits the high stresses with a slight decrease at the crack initiation location for all thicknesses. If the hydrostatic stresses are compared (which is the negative of the pressure plotted in Figure 6c, for all thicknesses the unclamped region is under positive hydrostatic stress except the zone underneath puncher for 1 mm. As the thickness gets smaller, the distribution of the hydrostatic stress becomes more uniform through the thickness. Obviously, this has direct consequences on the void nucleation and growth.

**Figure 7:**
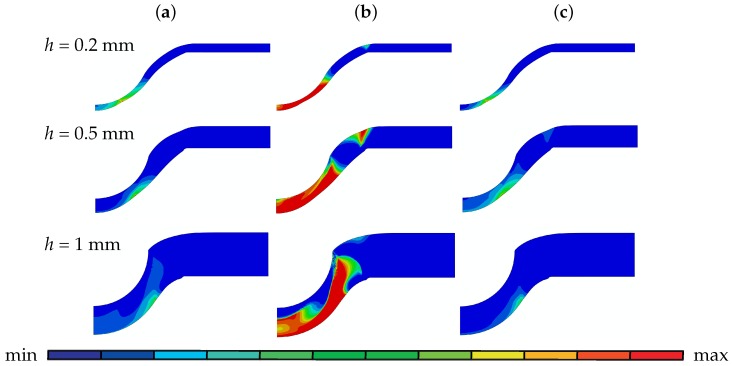
Damage plots (**a**) *f* (**b**) $f^{n}$ (**c**) $f^{g}$ of disks with 0.2 mm , 0.5 mm and 1 mm thickness before crack initiation: for *h* = 0.2 mm (**a**) [max $1.29\times 10^{-1}$; min $2.30\times 10^{-4}$], (**b**) [max $2.00\;\times \;10^{-2}$; min 0], (**c**) [max $1.08\times 10^{-1}$; min $-2.10\times 10^{-4}$], for *h* = 0.5 mm (**a**) [max $1.92\times 10^{-1}$; min $3.43\;\times \;10^{-5}$], (**b**) [max $2.00\times 10^{-2}$; min 0], (**c**) [max $1.72\times 10^{-1}$; min $-5.96\times 10^{-4}$], and for *h* = 1 mm (**a**) [max $2.02\;\times \;10^{-1}$; min $6.21\times 10^{-5}$], (**b**) [max $2.00\times 10^{-2}$; min 0], (**c**) [max $1.81\times 10^{-1}$; min $-5.90\times 10^{-3}$].

Figure 7 depicts the damage fields: *f*, $f^{n}$, $f^{g}$. Since in this figure $k_{w}$ is taken as 0, there is no damage growth due to shear. Hence, *f* is equal to the summation of $f_{0}$, $f^{n}$ and $f^{g}$. If pressure plots in Figure 6c are taken into account, one can see that just before crack initiation, nucleation mostly reaches its maximum value of 0.02 at the region under positive hydrostatic stress. Hence, with plastification all the void nucleation sources are exhausted where the distribution is rather uniform over the positive hydrostatic stress region. With thinner specimens the source of positive hydrostatic stresses is the stretching behavior. Thus underneath the punch there is considerable nucleation. This is not the case for increased thicknesses, where under the punch since the indentation mode is dominant, high compressive stresses with this confinement allows neither nucleation nor positive growth. $f_{\mathrm{normal}}^{g}$ also occurs in the region where positive hydrostatic stress prevails and has its maximum value where plastic strain is also maximal. For the disk with 1 mm thickness, the zone underneath the contact region where hydrostatic stress is negative $f^{g}$ is also negative. Thus, in this zone void shrinkage takes place instead of void growth.

**Figure 8:**
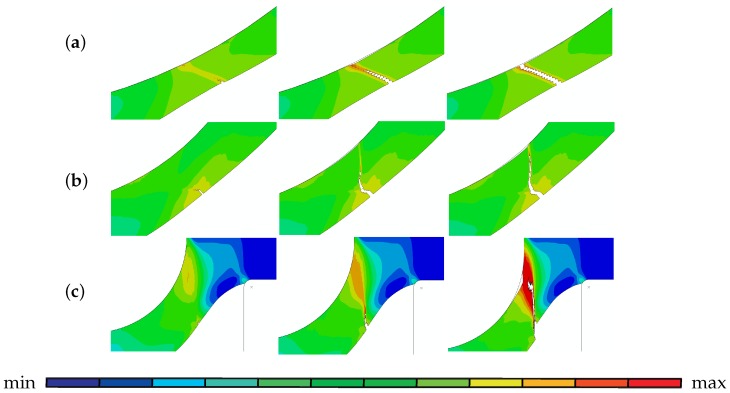
Initiation and propagation of the crack along the maximum plastic strain for disks with thicknesses of (**a**) 0.2 mm (**b**) 0.5 mm (**c**) 1 mm. For all cases min = 0, max = 1.5.

For all thicknesses, the crack initiates from the highest strained location underneath the puncher at the bottom surface and propagates upwards as shown in Figure 8, where plots of plastic strain are given in various stages of crack propagation for disks with thickness of 0.2 mm, 0.5 mm and 1 mm. This reveals the ductile crack propagation in the direction of the maximum plastic strain. Virtually maximum plastic strain occurs in the path due to localization of voidage and the crack simply follows this path since the main controlling variable for the crack path is the porosity. For the 0.2 mm disk, the final crack has an inclined path whereas for 0.5 mm disk the crack also starts with an inclined path but then kinks away almost half way. Similar kinking is also observed for this disk in the optical image of the section (see Figure 13c). As to the 1 mm disk, the crack initiating at the bottom side propagates vertically inside the shear band. Although there is a highly strained region at the upper side of the disk, both the maximum strain and damage localization occur at the bottom side where the crack initiates. In general, for thinner specimens the crack propagation direction is less inclined whereas for the thick ones, vertical crack propagation takes place. It is also seen that for the thin specimens relatively uniform fields are observed which is not the case for the thick ones.

#### 5.2.2. The Influence of Material Parameters

**Figure 9:**
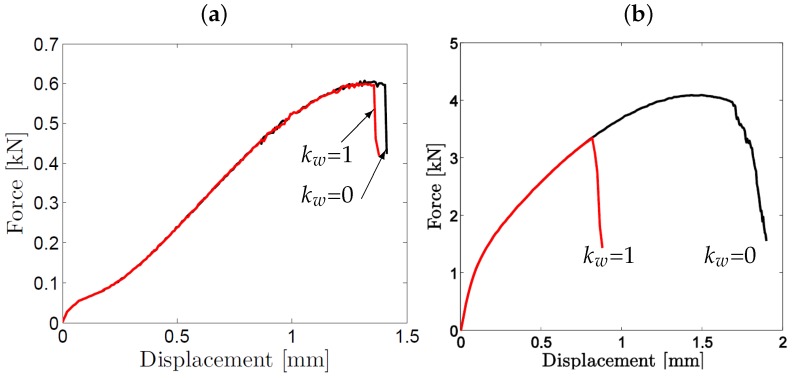
Effect of shear parameter, $k_{w}$ on force-displacement curves: disk thickness of (**a**) 0.2 mm and (**b**) 1 mm for values of $k_{w}$ = 0 and $k_{w}$ = 1.

The effect of shear softening due to void distortion and inter-void linking is investigated by using different values of the scaling parameter $k_{w}$ : $k_{w}=0$ and $k_{w}=1$ respectively representing no and full shear influence on ductile damage. These values are investigated for the two extreme thickness values: 0.2 mm and 1 mm. As concluded from Figure 9 increasing $k_{w}$ values results in an earlier loss of load carrying capacity, and thus, a decrease in the recorded displacements at incipient fracture. As anticipated, in the thinner disks the membrane stretching mode of deformation governs while shear effects are much less important. Accordingly, with an increase of thickness shear effects govern and as a consequence, the shear extension in the GTN model results in a considerable reduction in the materials ductility.

**Figure 10:**
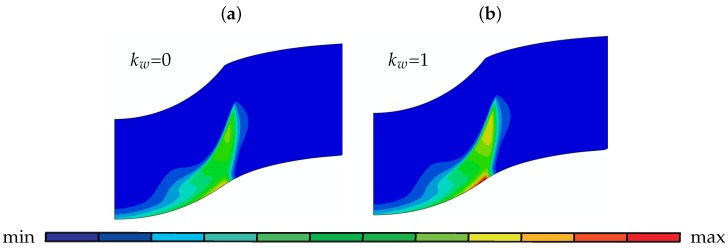
Damage development for a disk of 1 mm thickness at a displacement of 0.7 mm for (**a**) $k_{w}\;=\;0$ and (**b**) $k_{w}=1$: [max $2.02\times 10^{-1}$; min $6.21\times 10^{-5}$].

In Figure 10 the damage distribution plot at a displacement of 0.7 mm reveals how the $k_{w}$ parameter influences the total damage development in a 1 mm thick disk. In agreement with abovementioned observations, with the increase of $k_{w}$ from 0 to 1, the maximum damage value increases by 28% at the same displacement.

**Figure 11:**
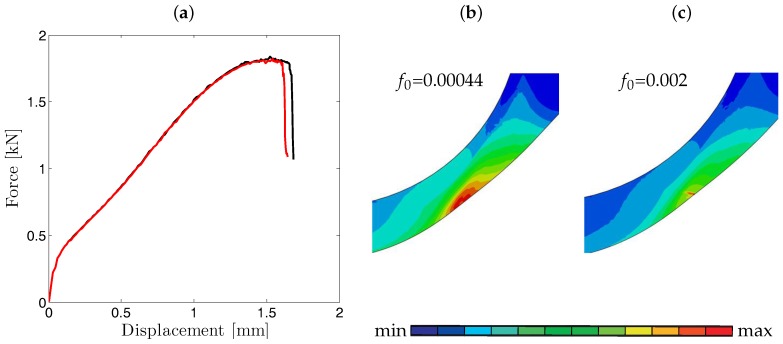
(**a**) Effect of initial porosity $f_{0}$ on force-displacement curves of 0.5 mm disk. The red curve represents $f_{0}=0.002$ whereas the black curve represents $f_{0}=0.00044$. Damage development for (**b**) $f_{0}=0.002$ [max $1.08\times 10^{-1}$; min $3.44\times 10^{-5}$] (**c**) $f_{0}=0.00044$ [max $1.81\times 10^{-1}$; min $1.51\times 10^{-4}$] at punch displacement of 1.6 mm.

The effect of $f_{0}$ on force-displacement curves is investigated by taking $f_{0}$ as 0.002 from [29] and 0.00044 calculated from Franklin’s formula. As anticipated, increased value of $f_{0}$ results in earlier fracture, while the hardening part of the curve is almost not affected (see Figure 11a). In Figure 11b,c the damage development of a 0.5 mm thick disk prior to crack initiation for $f_{0}=0.002$ and $f_{0}=0.00044$ are presented showing that with higher initial porosity the damage accumulation also increases.

In conclusion, if the shear extension is utilized, a premature fracture occurred, so the $k_{w}$ parameter had to be taken as 0 to achieve agreement with experimental results. As to the initial porosity, while the effect on force-displacement curves was not so prominent, the $f_{0}=0.00044$ as calculated from Franklin’s formula gave closer agreement with the experimental curve. Additionally, SP tests for largest and smallest thicknesses with and without damage effects were simulated to see its effect on necking. In all cases it is observed that necking occurs with or without damage whereas with damage its evolution with thinning until the final rupture occurs much faster.

#### 5.2.3. Comparison to Experimentally Determined Results

**Figure 12:**
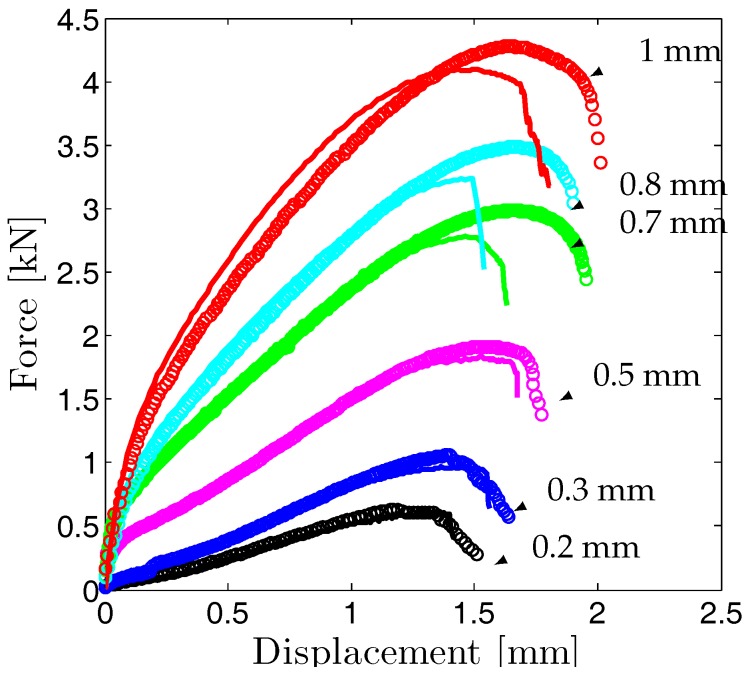
Comparison of experimental and computational force-displacement curves of disks of varying thicknesses with the parameter set in Table 1. Discrete circles represent the experimental data and the solid lines represent the simulations.

In Figure 12 the computational and experimental load-displacement curves of SP disks are compared. Experimentally two to three tests were carried out for each thickness. For comparison with the numerical curves, averages of experimental load-displacement curves are used. The results show a good agreement between computational and experimental curves especially in terms of hardening. The maximum strengths are slightly underestimated for disks with 0.7 mm, 0.8 mm and 1 mm thickness. Generally speaking, the calculated curves exhibited a steeper force drop compared to the experiments due to axisymmetry assumption in the simulations which prevails in both localization and fracture behaviour. It should be noted that for the selected sample size, shape, loading conditions and selected mesh size/length scale ratio both local and nonlocal formulation estimations were similar. The similarity was revealed in terms of force displacement plots, i.e., energy dissipation during fracture, fracture patterns and was attributed to the milder stress and strain gradients. Considering the severe change in element aspect ratios under stretching during SP test simulation, only nonlocal formulations supply spatially invariant material length. With this property, the developed framework constitutes a unified modeling environment for problems involving sharp notches where high stress gradients are evident or problems with smaller specimen sizes where size effects will govern.

**Figure 13:**
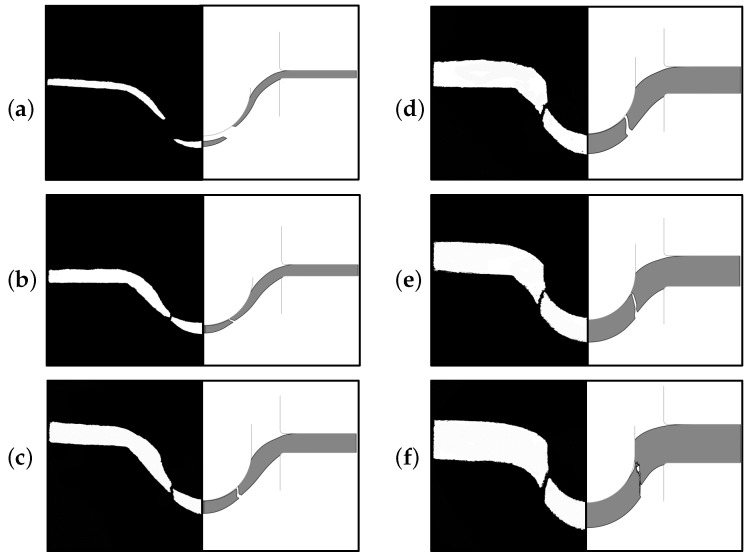
Comparison of experimental (left) and computational (right) deformed sections of SP disks of varying thickness: (**a**) *h* = 0.2 mm; (**b**) *h* = 0.3 mm; (**c**) *h* = 0.5 mm; (**d**) *h* = 0.7 mm; (**e**) *h* = 0.8 mm; (**f**) *h* = 1 mm.

In Figure 13 a comparison of the deformed profiles obtained from optical microscopy observations (left) and simulations (right) is given. The fractured SP disks are sectioned by a precision cutter and images are obtained by an optical microscope. Both crack locations and the paths of the cracks of the computational results are found to be in good agreement with the experimental ones. It is noted both from FE results and optical images that the location of crack initiation slightly moves further away from the center of the disk with increasing thickness.

**Figure 14:**
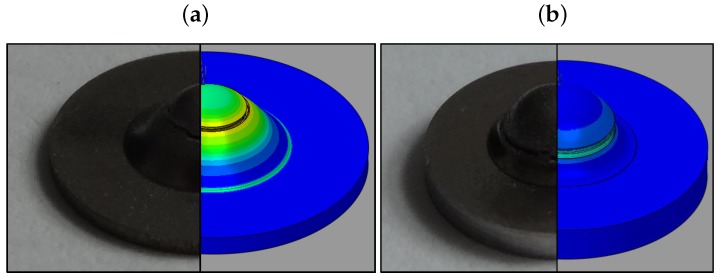
Circular crack path of disks with thicknesses of (**a**) 0.5 mm (**b**) 1 mm: optical images on the left and simulation on the right.

In 3D, the crack follows approximately a circular path along the maximum plastic strain contour for all thicknesses (see Figure 14) which complies with the axisymmetry assumption applied in the present modeling.

## 6. Conclusions and Outlook

In this study, a combined experimental and computational investigation of deformation and fracture during room temperature SP tests of P91 steel disks has been presented. To this end, tests were conducted on disks with different thicknesses. A void driven ductile fracture mode is recorded for all cases which is preceded by a diffuse necking with membrane stretching followed by a localized deformation for smaller thicknesses and shear localization for larger ones.

On the computational side a rate dependent porous-plastic constitutive model with a non-local extension is established to predict the deformation and fracture behavior of P91 steel during SP testing. The nonlocal formulation allows a natural control of the localization size through incorporating a material length parameter relating to the inter-particle spacing. With the developed framework besides a detailed full field investigation of the deformation process in the SP test, parameter sensitivity analyses are also realized. The set of material parameters were identified through a combined quantitative metallurgical and inverse mechanical analysis by comparison of the numerical and experimental force-displacement curves. The accuracy of the simulation results with the identified parameters were assessed by comparing experimental and computational force-displacement curves as well as the deformed sections of disks obtained from FE and optical images. A good agreement is captured between the experimentally and computationally determined force-displacement curves as well as the deformed sections at fracture.

The parameter sensitivity analysis conducted for the shear damage parameter $k_{w}$ shows that increasing values of $k_{w}$ result in earlier fracture and this effect got more pronounced with increasing disk thickness due to the influence of shear. To a lesser extent initial porosity, $f_{0}$, also affected the force-displacement curves in the same way since with higher values of $f_{0}$ the damage development was higher. The developed framework allows modeling ductile fracture in the presence of sharp stress gradients driven by, e.g., notches. The model was also found to be efficient in predicting the deformed geometry: necking patterns, crack initiation location and the crack propagation paths by comparing the sections of the failed samples obtained through simulations and optical microscopy. 

## Figures and Tables

**Table 1:** Flow curve data of P91 at room temperature.

$\sigma_{y0}$ [MPa]	$\sigma_{y1}$ [MPa]	$\sigma_{y\infty }$ [MPa]	$h_{0}$ [MPa]	$h_{1}$ [MPa]	*m* [-]	*n* [-]	$e_{0}^{p}$ [-]
520	376	831	123.31	75	6.14	0.541	0.006

## Supplementary Materials

The following are available online at www.mdpi.com/1996-1944/10/10/1185/s1.

## Abbreviations

The following abbreviations are used in this manuscript:

SP	small punch
GTN	Gurson-Tvergaard-Needleman
RKR	Ritchie-Rice-Knott
YS	yield stress
UTS	ultimate tensile strength
SEM	scanning electrone microscope
