# Supplementary material for: Experimental and Computational Study of Ductile Fracture in Small Punch Tests
Source: Materials (Basel). 2017 Oct 17;10(10):1185. doi: 10.3390/ma10101185 (PMC5666991; doi:10.3390/ma10101185)
Supplement: Supplementary file 1 [file materials-10-01185-s001.pdf]

Supplementary Materials: Experimental and Computational Study of Ductile Fracture in Small Punch Test

Betül Gülçimen [1], Celal Soyarslan [2*], Swantje Bargmann [3] and Peter Hähner [4]

0. Numerical Implementation

The complete set of equations to be solved can be reiterated as follows,

$$\left.\begin{array}{rcl} \dot{\hat{\epsilon}}^e & = & \dot{\hat{\epsilon}} - \dot{\hat{\epsilon}}^p\,, \\ \dot{\hat{\epsilon}}^p & = & \dot{\gamma}\partial_{\hat{\sigma}}\Phi^p\,, \\ \dot{\hat{\sigma}} & = & \mathbb{C}^e : \dot{\hat{\epsilon}}^e\,, \\ \dot{e}^p & = & \dot{\gamma}\boldsymbol{\eta} : \partial_{\hat{\sigma}}\Phi^p\,, \\ \dot{f} & = & \dot{\gamma}\left[A_N\boldsymbol{\eta} + \mathbf{B}_G\right] : \partial_{\hat{\sigma}}\Phi^p\,. \end{array}\right\} \tag{1}$$

$\boldsymbol{\eta} := \hat{\sigma} / \left[[1-f]\,\sigma_y\right]$ and $\mathbf{B}_G = \mathbf{B}_G\left(f, \operatorname{dev}\hat{\sigma}\right)$ is defined as

$$\mathbf{B}_G := [1-f]\,\mathbf{1} + k_w f \frac{w\left(\operatorname{dev}\hat{\sigma}\right)}{\sigma_{eq}} \operatorname{dev}\hat{\sigma}\,. \tag{2}$$

For solving Eqs. (1), an elastic predictor−plastic corrector type of algorithm is used. Letting $\Delta(\bullet) = \Delta t \times (\dot{\bullet})$, the subscript $n+1$ denote the (unknown) step at time t_{n+1} and n denote the (known) step at time t_n, the solution $\left\{\hat{\sigma}_{n+1}, e^p_{n+1}, f_{n+1}\right\}$ is sought for the given $\left\{\hat{\mathbf{T}}_n, e^p_n, f_n\right\}$ and the strain increment $\Delta\hat{\epsilon}$ with $\Delta t = t_{n+1} - t_n$. The corresponding operator-split is summarized in Table S1.

Table S1. Elastic predictor−plastic corrector type operator split.

Total		Elastic predictor		Plastic corrector
$\left\{\begin{array}{lcl} \Delta\hat{\epsilon} & \neq & \mathbf{0} \\ \Delta\hat{\epsilon}^p & \neq & \mathbf{0} \\ \Delta\hat{\sigma} & \neq & \mathbf{0} \\ \Delta e^p & \neq & 0 \\ \Delta f & \neq & 0 \end{array}\right\}$	=	$\left\{\begin{array}{lcl} \Delta\hat{\epsilon} & \neq & \mathbf{0} \\ \Delta\hat{\epsilon}^p & = & \mathbf{0} \\ \Delta\hat{\sigma} & = & \mathbb{C}^e : \Delta\hat{\epsilon} \\ \Delta e^p & = & 0 \\ \Delta f & = & 0 \end{array}\right\}$	+	$\left\{\begin{array}{lcl} \Delta\hat{\epsilon} & = & \mathbf{0} \\ \Delta\hat{\epsilon}^p & \neq & \mathbf{0} \\ \Delta\hat{\sigma} & = & -\mathbb{C}^e : \Delta\hat{\epsilon}^p \\ \Delta e^p & \neq & 0 \\ \Delta f & \neq & 0 \end{array}\right\}.$

Elastic Predictor

Here, a *trial* step is realized assuming the strain increment $\Delta\hat{\epsilon}$ is purely elastic. Once the corresponding value of the flow potential $\Phi^{p,trial}_{n+1}$ is smaller than zero, i.e. $\Phi^{p,trial}_{n+1} < 0$, the trial step is assumed to be correct, otherwise a plastic correction is required.

Plastic Corrector

The semi-implicit plastic corrector algorithm relies on exploitation of the first order Taylor series expansion of the yield potential around a known step $\langle i \rangle$

$$\Phi^{p\langle i+1\rangle}_{n+1} \approx \Phi^{p\langle i\rangle}_{n+1} + \mathbf{r}^{\langle i\rangle}_{n+1} : \delta\hat{\sigma}^{\langle i\rangle}_{n+1} + \xi^{\langle i\rangle}_{n+1}\delta e^{p\langle i\rangle}_{n+1} + \varsigma^{\langle i\rangle}_{n+1}\delta f^{\langle i\rangle}_{n+1} + \varpi^{\langle i\rangle}_{n+1}\delta\dot{e}^{p,\langle i\rangle}_{n+1}\,, \tag{3}$$

where

$$\left.\begin{array}{rcccl} \mathbf{r} & := & \partial_{\hat{\sigma}}\Phi^p & = & \partial_{\sigma_{eq}}\Phi^p\partial_{\hat{\sigma}}\sigma_{eq} + \partial_{\sigma_m}\Phi^p\partial_{\hat{\sigma}}\sigma_m, \\ \xi & := & \partial_{e^p}\Phi^p & = & \partial_{\sigma_y}\Phi^p\partial_{e^p}\sigma_y, \\ \varsigma & := & \partial_f\Phi^p & = & 2q_1\cosh\left([3/2]\left[q_2\sigma_m/\sigma_y\right]\right) - 2fq_3, \\ \varpi & := & \partial_{\dot{e}^p}\Phi^p & = & \partial_{\sigma_y}\Phi^p\partial_{\dot{e}}\sigma_y\,. \end{array}\right\} \tag{4}$$

The increments $\delta\left(\bullet\right)^{\langle i\rangle} = \left(\bullet\right)^{\langle i+1\rangle} - \left(\bullet\right)^{\langle i\rangle}$ in (3) read

$$\left.\begin{array}{rcl}\delta\widehat{\boldsymbol{\sigma}}^{\langle i\rangle}_{n+1} & = & -\delta\gamma^{\langle i\rangle}_{n+1}\mathbb{C}^{e} : \mathbf{r}^{\langle i\rangle}_{n+1},\\ \delta e^{p\langle i\rangle}_{n+1} & = & \delta\gamma^{\langle i\rangle}_{n+1}\boldsymbol{\eta}^{\langle i\rangle}_{n+1} : \mathbf{r}^{\langle i\rangle}_{n+1},\\ \delta f^{\langle i\rangle}_{n+1} & = & \delta\gamma^{\langle i\rangle}_{n+1}\left[A^{\langle i\rangle}_{N,n+1}\boldsymbol{\eta}^{\langle i\rangle}_{n+1} + \mathbf{B}^{\langle i\rangle}_{G,n+1}\right] : \mathbf{r}^{\langle i\rangle}_{n+1},\\ \delta\dot{e}^{p,\langle i\rangle}_{n+1} & = & \delta e^{p\langle i\rangle}_{n+1}/\Delta t\,.\end{array}\right\} \tag{5}$$

Using the condition $\Phi^{p\langle i+1\rangle}_{n+1} = 0$ as required, and substituting (3) into the right-hand side of (5) which allows factoring out the incremental plasticity parameter, we find $\delta\gamma^{\langle i\rangle}_{n+1}$ as

$$\delta\gamma^{\langle i\rangle}_{n+1} = \frac{\Phi^{p\langle i\rangle}_{n+1}}{\mathbf{r}^{\langle i\rangle}_{n+1} : \mathbb{C}^{e} : \mathbf{r}^{\langle i\rangle}_{n+1} + \mathbf{r}^{\langle i\rangle}_{n+1} : \mathbf{D}^{\langle i\rangle}_{n+1}}\,, \tag{6}$$

where

$$\mathbf{D}^{\langle i\rangle}_{n+1} = \left[\xi^{\langle i\rangle}_{n+1} + \frac{\varpi^{\langle i\rangle}_{n+1}}{\Delta t}\right]\boldsymbol{\eta}^{\langle i\rangle}_{n+1} + \varsigma^{\langle i\rangle}_{n+1}\left[A^{\langle i\rangle}_{N,n+1}\boldsymbol{\eta}^{\langle i\rangle}_{n+1} + \mathbf{B}^{\langle i\rangle}_{G,n+1}\right]. \tag{7}$$

We start the iterations by assigning an initial guess to the plastic multiplier $\Delta\gamma^{\langle 0\rangle}_{n+1}$. This depends on the rate dependence of hardening which is assumed to vanish for $\dot{e}^{p} < \dot{e}^{p}_{0}$, that is $r_y = 1$ as $\dot{e}^{p} < \dot{e}^{p}_{0}$. Consequent numerical difficulty pertaining to the hardening discontinuity is remedied following in the lines of [1]. Consequently, once $\Phi^{p}\left(\Delta t \times \dot{e}^{p}_{0}\right) > 0$ we use the initial guess $\Delta\gamma^{\langle 0\rangle}_{n+1} = \Delta t \times \dot{e}^{p}_{0}$, otherwise $\Delta\gamma^{\langle 0\rangle}_{n+1} = 0$. State variable updates $\left(\bullet\right)^{\langle i+1\rangle} = \left(\bullet\right)^{\langle i\rangle} + \delta\left(\bullet\right)^{\langle i\rangle}$ are continued throughout the iterations $\langle i\rangle$ for the computed increment of the plastic multiplier in (6), until $\Phi^{p\langle i+1\rangle}_{n+1} \approx 0$ with a desired accuracy. The stress tensor is then rotated back to the current coordinates viz $\boldsymbol{\sigma}_{n+1} = \mathbf{R}_{n+1}\cdot\widehat{\boldsymbol{\sigma}}_{n+1}\cdot\mathbf{R}^{\top}_{n+1}$.

1. Verification of Implementation through Benchmark Problems

The verification of the implementation is done using the benchmark studies presented in [2], where the problems involve uniform field tests conducted on a single finite element with side length of 1 mm. The first problem uses Gurson's model without shear extension which agrees with the solution of the current framework for k_w=0. The second problem compares numerical solutions with analytically handled results for different k_w values.

1.1. Dilatation

Dilatation in three directions is supplied by loading three faces of a cube by 0.01 m/s in normal direction while the other three faces are let stationary. In addition, all faces are given expansion free boundary conditions. The elastic material parameters are selected as $E = 200$ GPa and ν =0.3. The elastic limit of the matrix material is defined by $\sigma_{y0} = 200$ MPa. A power law function $\sigma_y\left[Ee^{p}/\sigma_y\right]^{n}$ with $n = 0.1$ is supplied as the flow curve. Extended Gurson's model parameters are selected as $q_1 = q_2 = q_3 = 1$. The initial void volume fraction is taken as $f_0 = 0.005$. Strain dependent void nucleation parameters are taken as $e_N = 0.3$, $S_N = 0.1$ and $f_N = 0.04$. Coalescence parameters are chosen to be $f_c = 0.15$ and $f_f = 0.25$. To create a comparison basis with the ABAQUS implementation where shear extension does not exist, the shear parameter is set as $k_w = 0$. In Figure S1, comparisons are presented between ABAQUS built-in Gurson model (Keyword `*Porous Plasticity`) and current VUMAT implementation using the same input parameters. The results for the modified and original Gurson models are identical for uniform expansion as the figures reveal.

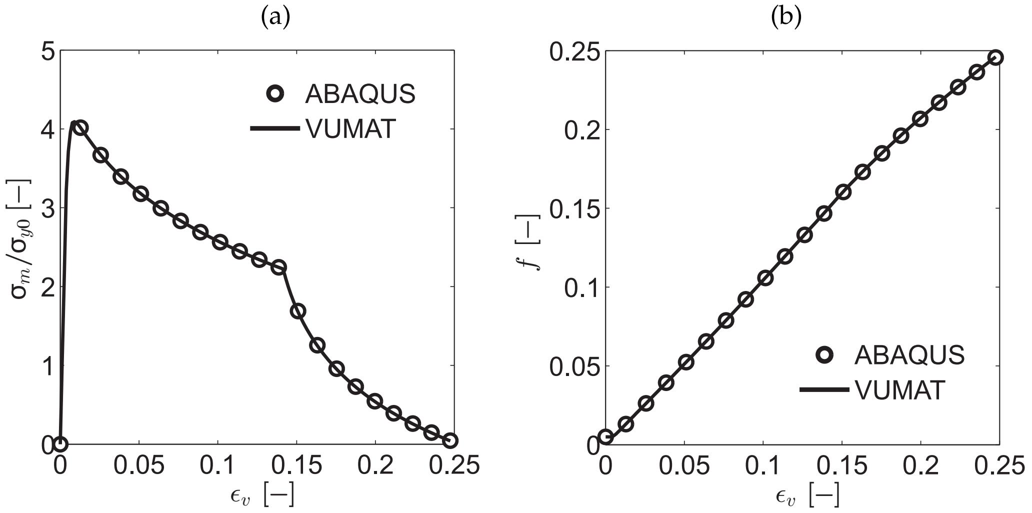

Figure S1. ABAQUS built-in model and VUMAT implementation comparisons for (**a**) σ_m/σ_{y0}, (**b**) void volume fraction histories during the dilatational loading.

1.2. Simple Shear

This problem is executed by excluding void nucleation and growth due to triaxiality and coalescence acceleration to facilitate a comparison with the following analytical solutions for f and σ_{eq} which neglect elasticity for simple shear in $(\mathbf{e}_1, \mathbf{e}_3)$ plane [2]

$$f = f_0 \exp\left(k_w e^p\right) \text{ and } \frac{\sigma_{eq}}{\sigma_{y0}} = \left[\frac{Ee^p}{\sigma_Y}\right]^n \left[1 - f_0 \exp\left(k_w e^p\right)\right] \tag{8}$$

The rest of the material parameters selected are identical to the previous problem. Simple shearing is supplied by loading one face with 0.01 m/s to obtain $\sigma = \tau\left[\mathbf{e}_1 \otimes \mathbf{e}_3 + \mathbf{e}_3 \otimes \mathbf{e}_1\right]$.

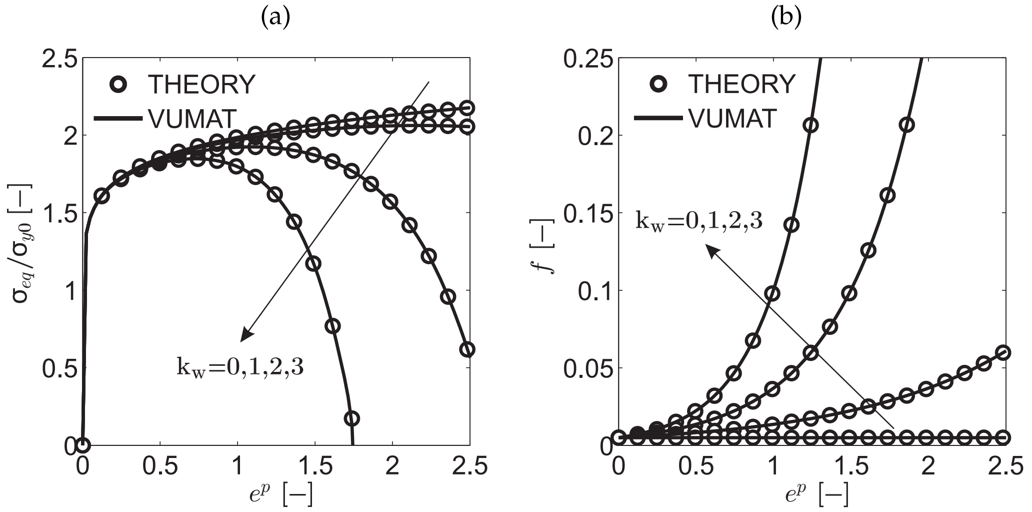

Figure S2. Analytical solution and VUMAT implementation comparisons for (**a**) σ_{eq}/σ_{y0}, (**b**) Void volume fraction histories during the shear loading.

As given in Figure S2 the resulting curves from the VUMAT implementations are in complete agreement with those of the analytical solution. As a conclusion, for $k_w > 0$ damage growth under shear stresses becomes exponential and increasing k_w reduces the localization and fracture strains that could

be reached. For $k_w = 0$ conventional Gurson's model response is carried out without an explicit dependence on shear.

2. Analysis of the Effectiveness of Delocalization

In order to verify the regularisation property of the developed nonlocal framework, plane strain tensile tests on imperfect models are realized. The imperfections are introduced as a smoothly distributed width change by 98% to the initially square domains with edges of 1 mm. Three cases with different element sizes $h = 0.05$ mm, $h = 0.025$ mm and $h = 0.0125$ mm are run. Thermal effects as an additional source of softening are switched off. The analysis is conducted for the ductile interaction radius of $R = 0$, which corresponds to the local analysis, and for $R = 0.15$ mm. As the contour plots for porosity development at the deformed configuration given in Figure S3 suggests, for the local analysis strong mesh dependence occurs as the mesh is refined a continuous reduction of the localization size results even at relatively low amounts of voidage which shows a clear loss of uniqueness. On the simulation results accounting for nonlocality however, it is seen that the localization band width as well as the magnitude of the maximum observed porosity could be kept constant. This verifies the desired delocalization and regularisation property of the developed framework.

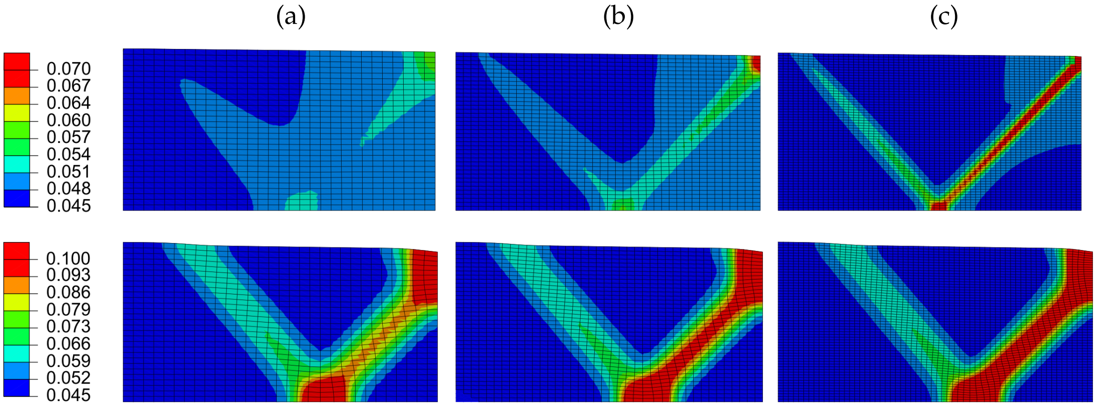

Figure S3. Total damage contours and localization patterns for local (top) and nonlocal (bottom) formulations for three different element sizes, (a) 0.05 mm, (b) 0.025 mm and (c) 0.0125 mm. The rows differ in investigated time steps since they represent different formulations whereas the columns do not.

References

1. Zaera, R.; Fernandez Saez, J. An implicit consistent algorithm for the integration of thermoviscoplastic constitutive equations in adiabatic conditions and finite deformations. *International Journal of Solids and Structures* **2006**, *43 (6)*, 1594–1612.
2. Nahshon, K.; Xue, Z. A modified Gurson model and its applications to punch-out experiments. *Engineering Fracture Mechanics* **2009**, *76*, 997–1009.
